# Cross-population analysis for functional characterization of type II diabetes variants

**DOI:** 10.1186/s12859-019-2835-0

**Published:** 2019-06-20

**Authors:** Dalia Elmansy, Mehmet Koyutürk

**Affiliations:** 1 Department of Electrical Engineering and Computer Science, Case Western Reserve University, 10900 Euclid Ave, Cleveland, OH 44106 USA; 2 Department of Electrical Engineering and Computer Science, Center for Proteomics and Bioinformatics, Case Western Reserve University, 10900 Euclid Ave, Cleveland, OH 44106 USA

**Keywords:** Cross-population analysis, Overlap analysis, Type II diabetes, T2D single nucleotide polymorphism, Functional annotation, Network analysis

## Abstract

**Background:**

As Genome-Wide Association Studies (GWAS) have been increasingly used with data from various populations, it has been observed that data from different populations reveal different sets of Single Nucleotide Polymorphisms (SNPs) that are associated with the same disease. Using Type II Diabetes (T2D) as a test case, we develop measures and methods to characterize the functional overlap of SNPs associated with the same disease across populations.

**Results:**

We introduce the notion of an Overlap Matrix as a general means of characterizing the functional overlap between different SNP sets at different genomic and functional granularities. Using SNP-to-gene mapping, functional annotation databases, and functional association networks, we assess the degree of functional overlap across nine populations from Asian and European ethnic origins. We further assess the generalizability of the method by applying it to a dataset for another complex disease – Prostate Cancer. Our results show that more overlap is captured as more functional data is incorporated as we go through the pipeline, starting from SNPs and ending at network overlap analyses. We hypothesize that these observed differences in the functional mechanisms of T2D across populations can also explain the common use of different prescription drugs in different populations. We show that this hypothesis is concordant with the literature on the functional mechanisms of prescription drugs.

**Conclusion:**

Our results show that although the etiology of a complex disease can be associated with distinct processes that are affected in different populations, network-based annotations can capture more functional overlap across populations. These results support the notion that it can be useful to take ethnicity into account in making personalized treatment decisions for complex diseases.

## Background

Genetic variations constitute an important part of the factors that contribute to many complex diseases. To identify genetic variations that are associated with specific complex diseases, Genome-wide and whole-genome association studies have been widely performed in recent years. These studies have identified many germline variants (in particular, single nucleotide polymorphisms or SNPs) associated with complex diseases in many different populations. Most of these identified variants exhibit subtle disease associations, and these variants usually have limited predictive power in risk assessment [1].

While the effect of a single genetic variation, like a SNP, could be small or large, the collective effect of many variations, and their relationships, provide valuable information into the mechanisms of complex diseases. To this end, there is an eminent need for studying the collective effect of many SNPs and their interrelationships as we try to characterize the functional underpinnings of the relationship between genotype and phenotype. One useful source of information in this regard is the disease associations identified by GWAS on different populations.

Since many genomic variants can be population-specific and their distribution can follow geographical patterns, GWAS on different populations offer different [2] and potentially complementary disease associations, which may enrich our understanding of disease mechanisms. Furthermore, rare variants, which are often thought to be the causal variants for many complex diseases, are usually population-specific [1]. Therefore, elucidation of the functional relationship between rare variants identified in different populations can be useful for the design of personalized treatment strategies in precision medicine. In the future, the comprehensive knowledge we collect from the use of multiple populations in whole-genome association studies can be utilized by healthcare systems in smoothing out and gradually eliminating health disparities [3, 4].

Systems biology uses computational and mathematical approaches to model the complex interactions among genetic variations as related to a phenotype. Such a holistic approach of characterizing and integrating the widespread genetic variations and the interplay between them yields a more intuitive understanding of complex diseases. Type II Diabetes (T2D) is one of the complex diseases, where most disease-associated variants identified by GWAS are different across different populations. Since variants identified on different populations can be linked to T2D through similar functional mechanisms, trying to annotate the significant SNPs from each population separately will not fully utilize the information obtained from different populations. Furthermore, since there are distinct underlying disease processes and treatment regimens for T2D, analyzing the functional overlap between variants identified on different populations can shed light into the differences between different populations in terms of disease mechanisms.

In this paper, we propose a computational pipeline to systematically assess the functional overlap between genomic markers of complex diseases that are identified on different populations. For this purpose, we use T2D as a suitable model disease, and we compile results from GWAS that have been performed on 9 different populations across the globe. These results mainly represent genomic loci that are found to be associated with T2D on samples collected from these nine populations. Interestingly, there is little overlap between disease associated loci identified on different populations.

**Fig. 1:**
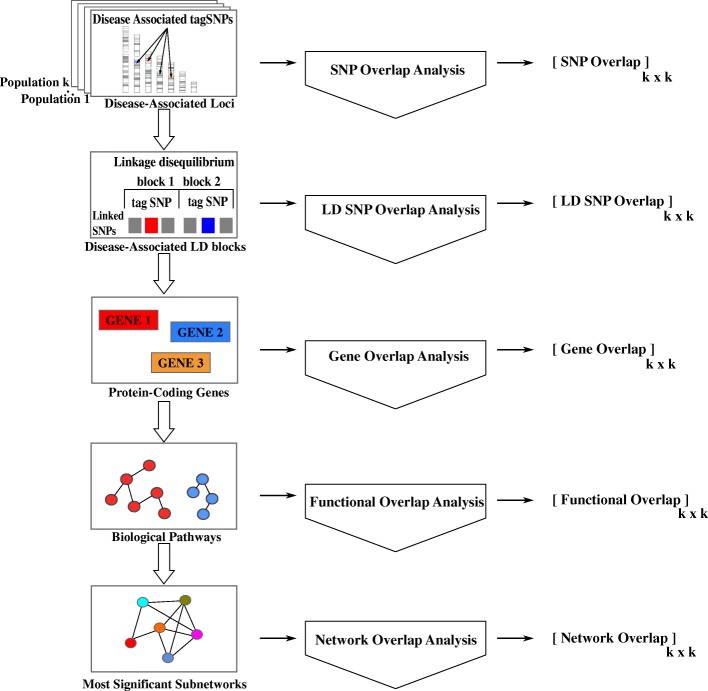
Workflow of multi-level functional overlap analysis for disease-associated genomic variants identified in different populations. The computational pipeline takes as input a set of genomic loci that are found to be significantly associated with a disease of interest in each of the populations that are considered. It then maps these loci to functional elements with coarser granularity, first considering linkage disequilibrium, then mapping loci to protein-coding genes, subsequently mapping these genes to biological pathways, and finally identifying functionally associated subsets of these genes via network analysis. At each level, the resulting functional elements that are found to be associated with the disease are compared across populations, to systematically characterize the overlap between different populations

To systematically assess the functional overlap between these different sets of SNPs, we develop computational algorithms and statistical frameworks, expecting that the variants identified in certain populations correspond to similar biological processes. For this purpose, we develop a multi-layered framework, where genomic loci, protein-coding genes, biological pathways in which these proteins are active, and networks of physical and functional interactions between these proteins are systematically evaluated for potential overlap**.** Figure 1 illustrates the framework. Our results show that the overlap between different populations grow as the level of abstraction coarsens from genomic location to biological function. More interestingly, our results also show that differences in the biological processes that are implicated in different populations align with the targets of T2D first-line therapy in each population.

To further assess the generalizability of our methods, we repeat the same pipeline of analyses on datasets for another complex disease, prostate cancer as a second test case. Our results on prostate cancer also show that more functional overlap can be detected among populations as the level of abstraction coarsens.

## Results

### Populations and datasets for type II diabetes

We use nine T2D Genome Wide Association datasets representing nine populations of Asian and European ethnic origins. The basic statistics of these studies and their results are shown in Table 1. The first two datasets are case-control studies for which the genotypes for all samples and genotyped loci are available. The other seven datasets are published results of case-control studies. These datasets consist of the list of significant loci that are identified by the study and the associated statistical significance figures.

**Table 1:** Descriptive statistics of the T2D studies, datasets, and results used in this study. The letter code shows the population from which the samples were obtained, Significant tagSNPs shows the number of tagSNPs that were found to be significantly associated with T2D at the significance threshold applied by the corresponding study (also shown in the table). Significant tagSNPs+LD shows the total number of these significant SNPs and the number of SNPs that are in linkage disequilibrium with these SNPs, but were not screened by the corresponding study

Populations	# Cases	# Controls	# Screened SNPs	# Significant tagSNPs	Significance Threshold (P-value)	# Significant tagSNPs + LD
British (W)	1,999	1,504	495,477	482	< 1e −5	1,831
American + European (D)	1,007	983	561,656	350	< 1e −8	1,686
Finnish (F)	1,161	1,174	317,503	157	< 5 x1e − 7	898
French (Fr)	679	697	392,365	4	< 7 × 1e −4	71
Saudi (S)	2,207	2,276	48	41	< 0.05	297
Korean (K)	462	456	2,188,613	395	< 1e −5	1,859
Japanese (J)	23,399	31,722	7,521,072	211	< 1e −8	668
Chinese (C)	684	955	2,900,000	33	< 2.6 x 1e−8	361
Lebanese (L)	1,902	1,384	5,891,794	23	< 1e −5	61

The nine datasets are the following:

1. Wellcome Trust Case Control Consortium –WTCCC (W) T2D SNP data which has genotyped 495,477 SNPs genotyped for 1999 case and 1504 control samples obtained from the UK population [5]. The control samples represent individuals who were born in 1958.
2. The database of Genotypes and Phenotypes –dbGAP (D) which has genotyped 561,656 SNPs for 1007 case and 983 control samples obtained from other parts of Europe and US [6].
3. A Finnish (F) population case-control study which genotyped 329,091 SNPs for 1161 case and 1174 control samples collected in Finland [7, 8].
4. A French (Fr) population case-control study which has genotyped 392,365 SNPs for 679 case and 697 control samples collected in France [9].
5. Saudi (S) population studies that pooled 48 Saudi T2D SNPs implicated in previous experiments for a total of 2207 case samples and 2276 control samples collected in Saudi Arabia [10–12].
6. A Korean (K) population case-control study which has genotyped 2,188,613 SNPs for 462 case samples and 456 control samples collected in South Korea [13, 14].
7. A Japanese (J) population case-control study which has genotyped 7,521,072 SNPs for 23,399 case and 31,722 control samples collected in Japan [15, 16].
8. A Chinese (C) population case-control study which has genotyped 2,900,000 SNPs for 684 case and 955 control samples collected in China [17, 18].
9. A Lebanese (L) population case-control study which has genotyped 5,891,794 SNPs for 1902 case and 1384 control samples collected in Lebanon [19].

### Functional overlap among genomic variants found to be associated with T2D in different populations

In this section, we present the overlap between T2D-associated SNPs at five different functional levels. For each functional level, we compute (i) an overlap matrix and (ii) a cumulative overlap function. Each overlap matrix is a *k* × *k* matrix that represents the pairwise overlap between the disease-associated loci in pairs of populations based on a certain notion of functional overlap, where *k* is the number of populations. Each cumulative overlap function is a function in the form *f*:{1, *…, k*} → [0,1], assessing the fraction of biological entities (individual loci, loci in LD, genes, functions, subnetworks) that are found to be associated with the disease in at least a given number of the populations.

We hierarchically cluster the populations using each of the five overlap matrices and visualize the overlap matrices as heatmaps with hierarchical clustering. To assess the statistical significance of the overlap functions, we report the results of permutation tests obtained through 1000 permutations (the procedure we use for the permutation tests is described in Methods). We compare the overlap function computed on the original dataset against the distribution of overlap functions computed using permutation tests, representing one thousand simulated runs.

**Fig. 2:**
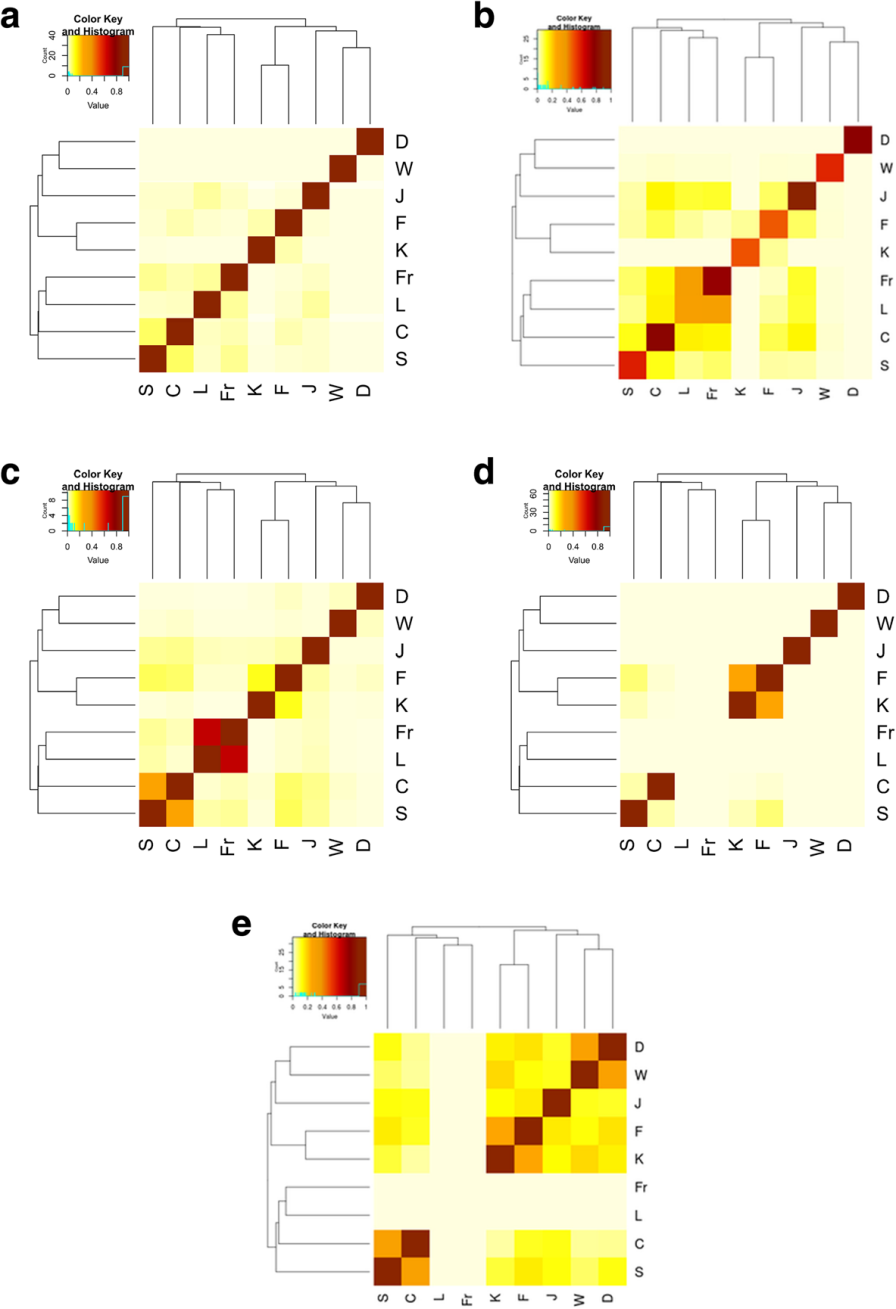
Overlap matrices at five functional levels for the genomic variants that are found to be associated with Type II Diabetes in 9 different populations. The plots from (a) to (e) show the overlap matrices for SNP, LD SNP, gene, functional and network overlap matrices respectively, depicted as heatmaps. Each heatmap has hierarchical clustering of the 9 populations (D = dbGAP, W=WTCCC, J = Japanese, F=Finish, K=Korean, Fr = French, L = Lebanese, C=Chinese, S=Saudi). The color intensity from white to brick, shows the degree of overlap, with brick being the highest

**Fig. 3:**
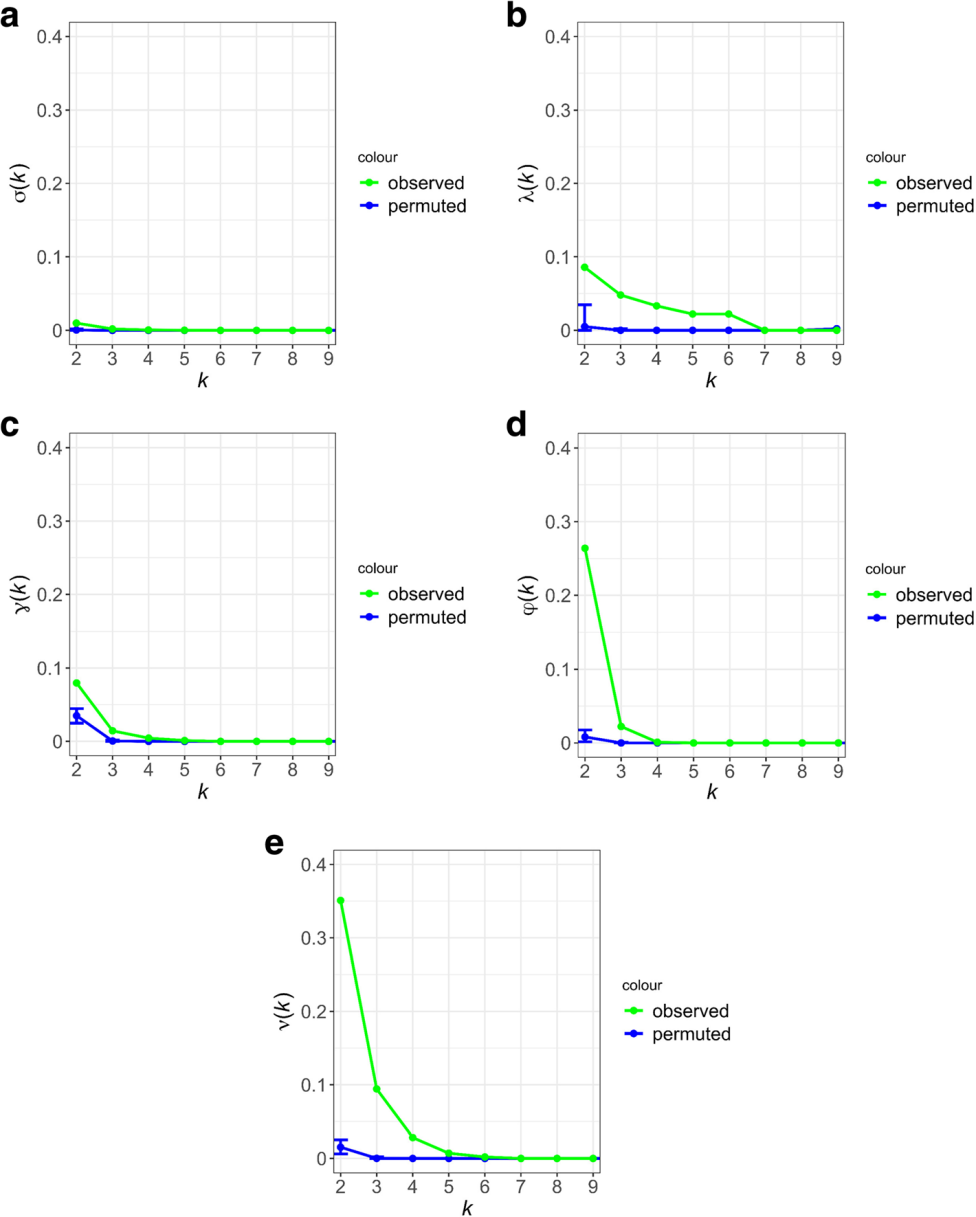
Cumulative overlap functions at five functional levels for the genomic variants that are found to be associated with Type II Diabetes in 9 different populations The plots from (a) to (e) show the SNP, LD SNP, gene, functional and network cumulative overlap functions respectively. Each plot also provides a comparison to the corresponding cumulative overlap function computed over 1000 random permutations

The SNP overlap matrix and the SNP overlap function for T2D-associated SNPs in the nine populations are shown in Fig. 2(a) and Fig. 3(a), respectively. As seen in the figures, the overlap between any pair of populations is considerably low, but there is some overlap between pairs of populations (Chinese and Saudi Arabian, French and Lebanese, Finnish and Korean, Lebanese and Japanese). Although the pairwise overlap between T2D-associated SNPs is considerably low, the permutation test for the overlap function for k = 2 (two populations) suggests that the pairwise overlap is statistically significant (z-score = 23.8, *p* < 3.82E-125). In other words, a SNP that is found to be associated with T2D in one population is likely to be found to be associated with T2D in at least one other population. However, for values of *k* larger than 2, the overlap between T2D-associated SNPs is not statistically significant, i.e. the T2D-associated SNPs do not tend to be shared across 3 or more populations.

The linkage disequilibrium (LD) SNP overlap matrix and the LD-SNP overlap function for T2D-associated SNPs in the nine populations are shown in Fig. 2(b) and Fig. 3(b), respectively. When we take linkage disequilibrium into account (thereby considering loci with correlated genotypes as overlapping), we observe considerably larger overlap between pairs of populations in the corresponding heatmap. In particular, the Lebanese and French populations cluster together with very high overlap. Another cluster is formed by the Chinese, Japanese, Finnish, and Saudi Arabian populations. This cluster also exhibits considerable overlap with the French-Lebanese cluster. The permutation test for the LD-SNP overlap function suggests statistically significant overlap (*p* < 4.27E-17) for up to 7 populations – thus we can conclude that, at such genomic level, there is some overlap between the T2D-associated loci among most of the populations that are considered.

The gene overlap matrix and the gene overlap function for T2D-associated genes in the nine populations are shown in Fig. 2(c), Fig. 3(c), respectively. Here, a gene is considered T2D-associated in a population if at least one SNP in the genes’ region of interest (ROI) is found to be significantly associated with T2D in that population. As seen in the heatmap, when genes are considered, more overlap is detected between pairs of populations, as compared to LD Analysis, e.g., there is solid pairwise overlap between populations like the Lebanese and French as well as the Chinese and Saudi Arabian. However, when more than two populations are considered, less overlap is detected in genes than in LD-SNPs – and no gene that is common to 6 populations is identified. This is shown by the distribution of the values of the overlap function for the permutation test, but the gene overlap is statistically significant for k = 2,3,4,5 populations (*p* < 1.1E-245).

The functional overlap matrix and the functional overlap function for T2D-associated functions in the 9 populations are shown in Fig. 2(d) and Fig. 3(d), respectively. As seen in the heatmap, there are no functions that are enriched in SNPs found to be associated with T2D in the French and Lebanese populations, so the two populations are eliminated from further analysis. There is an improved statistically significant pairwise overlap (z-score = 433.8, which corresponds to a very small *p*-value), between the Finnish and Korean populations, however the extent of overlap decreases to less than five populations as shown by the distribution of the values of the overlap function for the permutation test. In other words, a function that is found to be associated with T2D in one population is likely to be found to be associated with T2D in another three populations. However, for values of *k* larger than 4, the overlap between T2D-associated functions is not statistically significant i.e. the T2D-associated functions do not tend to be shared across 5 or more populations.

The network overlap matrix for T2D-associated subnetworks and the network overlap function for the seven populations (excluding Fr and L) are shown in Fig. 2(e) and Fig. 3(e), respectively. As seen in the heatmap, the amount and extent of overlap between populations is considerably higher than all previous overlap analyses. There is very high overlap between the Saudi and Chinese populations, populations of European ethnic origin represented by UK, US and other parts of Europe, and between the Finish and Korean populations. Moreover, there is highly statistically significant overlap (z-score > 1294, which corresponds to a very small *p*-value) between up to 6 populations. This is suggested by the distribution of the values of the overlap function for the permutation test. In other words, a subnetwork that is found to be associated with T2D in one population is likely to be found associated with T2D in almost all of the populations that are considered.

**Fig. 4:**
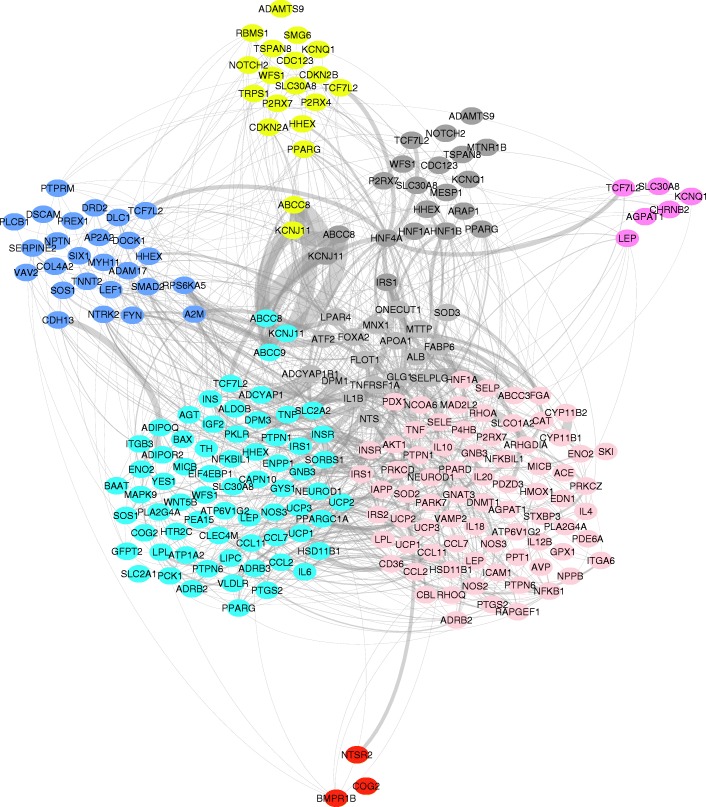
Cross-population protein-protein interaction network associated with Type II Diabetes. The proteins in the network are color-coded based on the population on which the corresponding gene is found to be associated with T2D. Blue:UK, Red:US+Other parts of Europe, Cyan:Finnish, Magenta: Korea, Purple:Japanese, Citric:Chinese, Dark Grey:Saudi, Light Grey is used for interconnection nodes. The edge width represents the interaction weight as calculated using Prix-Fixe network algorithm [20]

In order to visualize the functional overlap across the populations from a different perspective, we input the entire set of loci from all populations to the Prix-Fixe network analysis tool [20]. This tool outputs the most significant functionally coherent subnetwork across all seven populations. Subsequently, we plot the resulting protein-protein interaction (PPI) network, color coded for different populations. The T2D PPI network across seven populations is shown in Fig. 4. As shown in the figure, the resulting most functionally coherent subnetwork has some strong inter-population interactions between T2D-associated proteins and the strength of interaction, represented by the width of the edges, suggests a strong overlap between the Asian populations; specifically Chinese, Saudi, Korean, Japanese and Finnish populations and some overlap between the Finnish and British as well as between the American and other European and the Korean populations. This conforms to the previous results from network analysis.

### Correspondence between genes identified in different populations and T2D subtypes

**Fig. 5:**
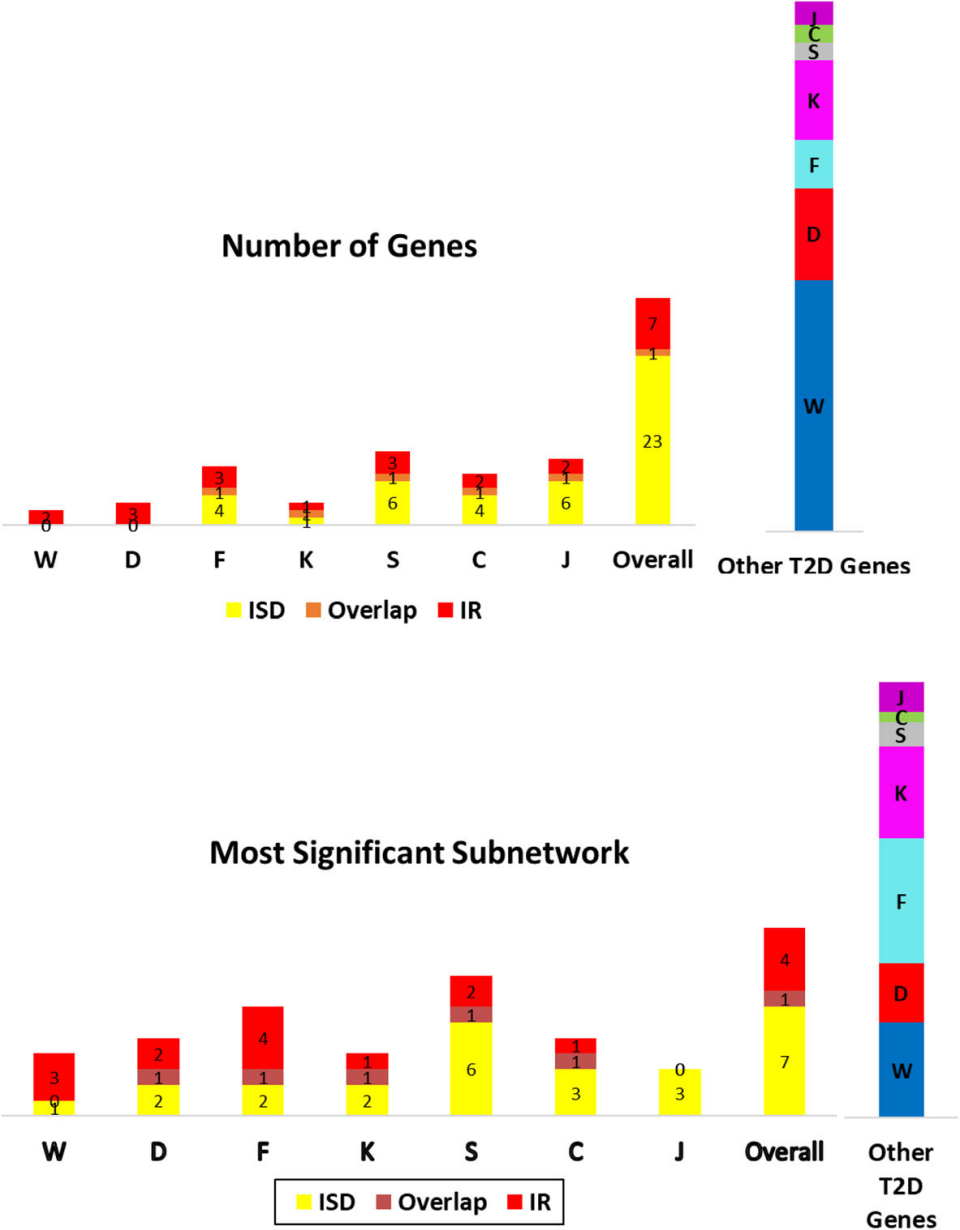
The correspondence between genes identified as associated with Type II Diabetes in different populations and their role in T2D subtypes. The top panel shows the distribution of the genes that are found to be associated with T2D in each population with respect to their association with two common T2D subtypes. For each population, among the genes that are found to be associated with T2D in that population, yellow bars represent the number of genes that are associated with Insulin Secretion Deficiency, red bars represent the number of genes associated with Insulin Resistance subtype and the orange bars represent the number of genes that are associated with both subtypes. The bottom panel shows the corresponding distribution for network-based overlap, where a gene is considered to be identified in a population if it is in the most significant subnetwork identified by network analysis on that population. The color codes used in the right panel are identical to that in the left panel. The rightmost bar shows the number of all other T2D genes that were identified in at least one population, color-coded for the population

According to Cantley J and Ashcroft [21], there are two main molecular mechanisms that underlie the etiology of T2D; insulin secretion deficiency and insulin resistance. Prasad and Groop [22] classify T2D associated genes according to their roles in these two subtypes. To investigate the correspondence between the genes that are found to be associated with T2D in each population and their association with these subtypes, we refined our analysis to the gene-based and network-based levels. The results of the analysis is shown in Fig. 5. In the figure, the number of genes that are found to be associated with T2D (based on the mapping of the variants identified in each population to the genes’ regions of interest) is stratified according to T2D subtypes. Since analysis at the network level provides system-level information, we also repeat this analysis at the network level and we observe increased overlap with network-based analysis. At the network level, we consider a gene to be identified in a population if the most significant subnetwork identified in that population contains the gene. Therefore, a gene that does not have a significant variant in a population can be found to be associated with T2D in that population, if it is functionally associated with other genes that harbor significant loci. Similarly, a gene that harbors a significant locus in a population may not be considered as associated with T2D at the network level, if its network neighborhood is not enriched in genes that are significant in that population.

As seen in Fig. 5, the genes identified in Asian populations are mostly associated with insulin secretion deficiency at both levels of genomic granularities, as opposed to European and American populations, in which insulin resistance seems to be the prevailing predisposing genetic factor for T2D, at both individual gene and network levels. For example, the US, UK, and other European populations have no insulin secretion-implicated genes at the genetic-level of the analysis, while the Finnish population shows an equal share of both at the genetic-level. However, at the network-level, all European populations including the Finnish population, are dominated by insulin resistance-implicated genes, while having some share of insulin secretion deficiency-related genes interacting with them. On the other hand, the Asian populations have more insulin secretion deficiency-implicated genes at the level of individual genes, except for the Korean population, which shows an equal share of insulin secretion deficiency and insulin resistance-implicated genes. At the network level, all Asian populations show more insulin secretion deficiency as opposed to insulin resistance, including the Korean population. Interestingly, none of the insulin resistance associated genes are identified to be associated with T2D in the Japanese population, according to the network level analysis**.**

### Application to prostate Cancer

In this section, we present the results obtained by applying the proposed framework to prostate cancer data. We use published results of Prostate Cancer Genome Wide Association Studies for seven populations representing seven ethnic origins. The basic statistics of these studies and their results are shown in Table 2. The seven datasets are the following:

**Table 2:** Descriptive Statistics for the genomic variants associated with Prostate Cancer. Significant tagSNPs shows the number of tagSNPs that were found to be significantly associated with Prostate Cancer at the significance threshold applied by the corresponding study (also shown in the table). Significant tagSNPs+LD shows the total number of these significant SNPs and the number of SNPs that are in linkage disequilibrium with these SNPs, but were not screened by the corresponding study

Populations	# Cases	# Controls	# Screened SNPs	# Significant tagSNPs	Significance Threshold (P-value)	# Significant tagSNPs+LD
European	90,843	72,487	> 10.5 M	161	< 10–8	9047
Hispanic	196	472	49	12	< 10–4	487
Japanese	6167	12,187	6,779,114	32	< 10–7	2487
Chinese	1912	1648	942,613	25	< 0.05	2178
Korean	1515	3189	60,276	10	< 10–7	644
African American	5869	5615	199	27	< 10–2	765
Tunisian	90	131	534,781	14	< 10–4	350

1. A European population represented by pooled results of European Prostate Cancer GWAS for more than 10.5 million genotyped SNPs and for a total of 90,843 case samples and 72,487 control samples of European ethnic origin [23–27].
2. A Hispanic population represented by the study of 49 haplotyped-tagged SNPs genotyped from 196 case and 472 control samples of Hispanic ethnic origin [28].
3. A Japanese population represented by the results of case-control studies which pooled a total of > 6,779,114 genotyped SNPs for a total of 6167 case and 12,187 control samples of Japanese ethnic origin [29, 30].
4. A Chinese population represented by the results of case-control studies which pooled a total of 942,613 genotyped SNPs for a total of 1912 case and 1648 control samples of Chinese ethnic origin [31–34].
5. A Korean population study that genotyped 60,276 SNPs from 1515 case and 3189 control samples of Korean ethnic origin [35].
6. An African American population study that genotyped 199 SNPs from 5869 case and 5615 control samples of African American ethnic origin [36].
7. A Tunisian population study that genotyped 534,781 SNPs from 90 case and 131 control samples of Tunisian men [37].

**Fig. 6:**
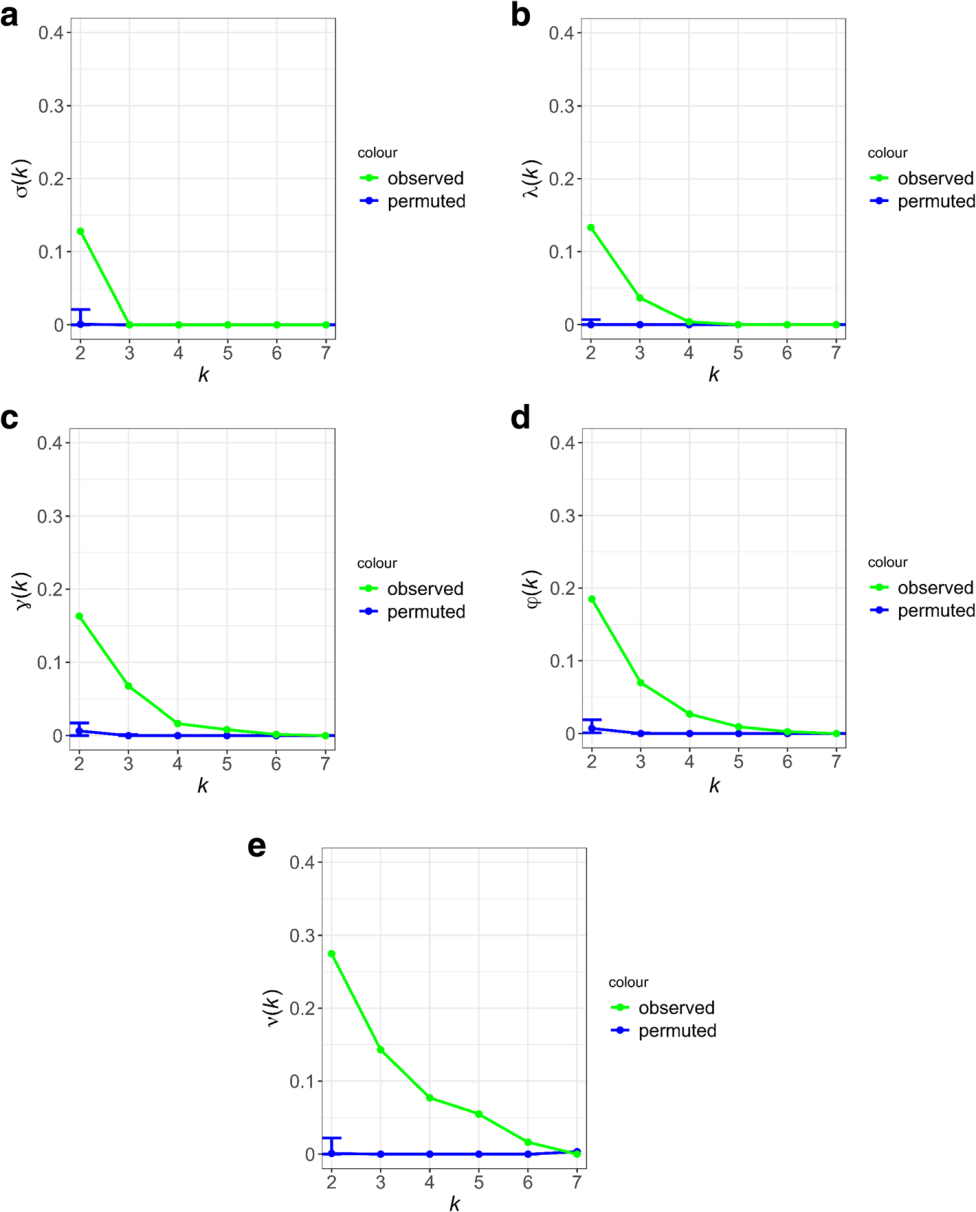
Cumulative overlap functions at five functional levels for the genomic variants that are found to be associated with Prostate Cancer in 7 different populations. The five cumulative overlap functions for prostate cancer, with increasingly statistically significant overlap as we go down the pipeline of analyses from SNP to Network Overlap analysis

We report the results of the five cumulative overlap functions assessing the fraction of biological entities (individual loci, loci in LD, genes, functions, subnetworks) that are found to be associated with prostate cancer in at least a given number of the populations. We compare the overlap function computed on the original dataset against the distribution of overlap functions computed using permutation tests, representing one thousand simulated runs (the procedure we use for the permutation tests is described in Methods). The results in Fig. 6(a-e) show that more significant overlap between populations is realized as the level of abstraction coarsens, from genomic location to biological function.

The permutation test for the overlap function for k = 2 (two populations) suggests that the pairwise overlap is statistically significant (Fig. 6(a), z-score = 43.151). In other words, a SNP that is found to be associated with prostate cancer in one population is likely be associated with prostate cancer in at least one other population. However, for values of *k* larger than 2, the overlap between prostate cancer associated SNPs is not statistically significant, i.e. the prostate cancer associated SNPs do not tend to be shared across 3 or more populations. In Fig. 6(b) the permutation test for the LD-SNP overlap function suggests statistically significant overlap for up to 3 populations (z-score = 281.63). In Fig. 6(c) the distribution of the values of the gene overlap function for the permutation test, suggests that the overlap is statistically significant for up to 3 populations (z-score = 489.521). In Fig. 6(d) the distribution of the values of the functional overlap function for the permutation test suggests that the overlap is statistically significant for up to 4 populations (z-score = 489.031) and in Fig. 6(e) the distribution of the values of the network overlap function for the permutation test, suggests that the overlap is statistically significant for up to 5 populations (z-score = 361.762).

## Discussion

According to literature [38, 39], most T2D treatment regimens are based on one of two groups of medications. The first of these two groups is Sulfonylureas; used to improve insulin secretion, by targeting the ABBC8 and KCNJ11 genes and the other is Metformin, which is used to improve insulin sensitivity and targets the PRKAB1 [40–43]. The drug targets for Sulfonylureas are implicated in the Asian populations of this study, as well as the Finnish population. The drug target for Metformin and the genes that interact with it (PRKAG2 and PRKAG1) are implicated in the European, American and the Korean populations.

**Fig. 7:**
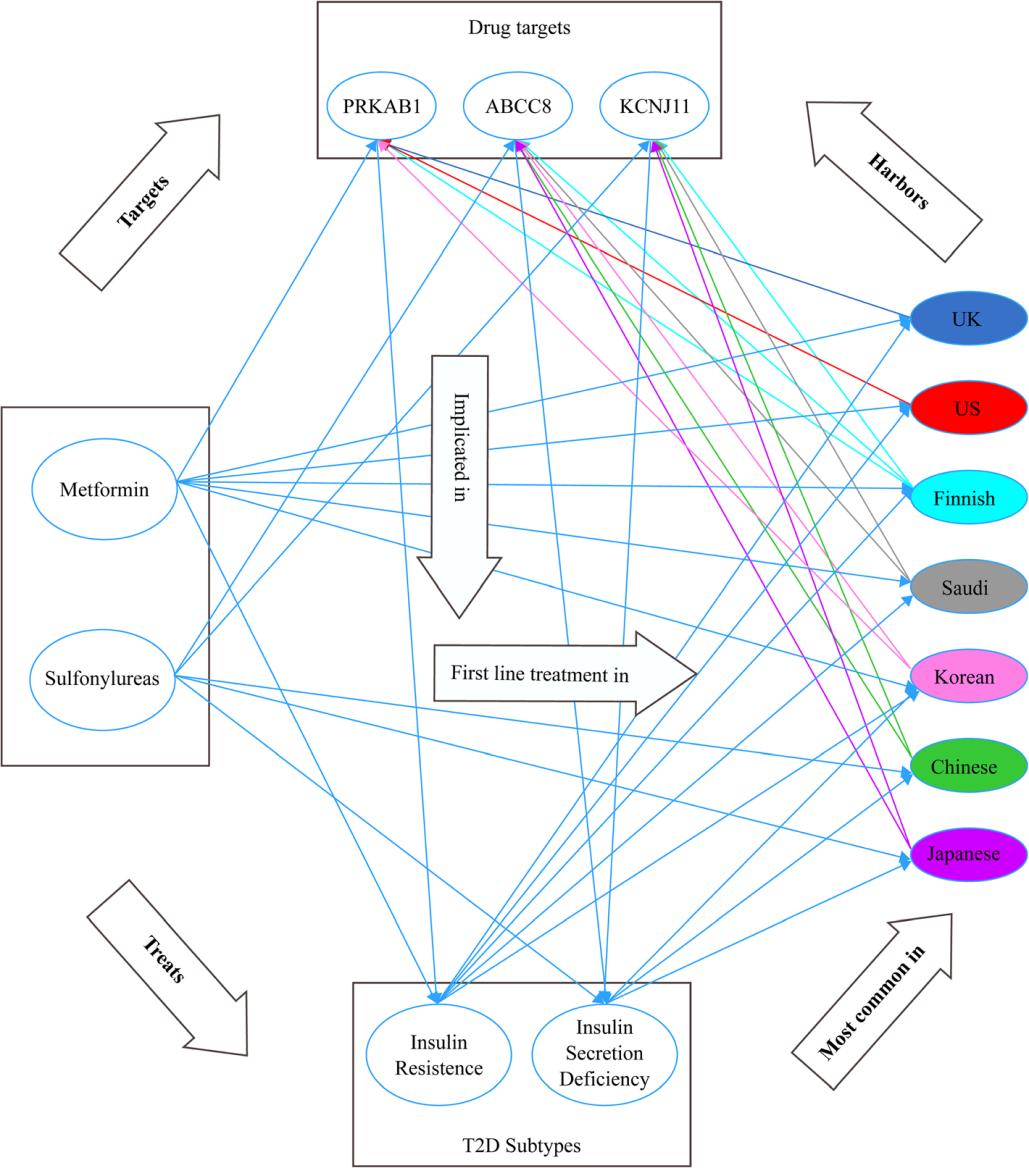
Interrelationship between subtypes, populations, first line treatments and drug targets for Type II Diabetes. The figure shows the triangular interrelationships between T2D subtypes, genes identified in each population in this study, first line T2D treatments in these populations, the drug targets of these first line treatments, and their implications in T2D subtypes. For example, in the US, insulin resistance is the most common T2D subtype and Metformin is the first-line T2D treatment. The drug target of Metformin is PRKAB1 which is implicated in insulin resistance and is harbored by the US population in this study

In Fig. 7, we show the interrelationship between T2D subtypes, genes identified in each population in this study, first line T2D treatments in these populations, the drug targets of these first line treatments, and their implications in T2D subtypes. It is interesting that the first line treatment for T2D in each of the populations in this study conform to the population’s unique T2D mechanisms. For example, literature confirms that decreased insulin secretion capacity takes a bigger role in the development of T2D in the Japanese population than insulin resistance. Furthermore, Sulfonylureas have been the most prescribed class of drugs, and has been the first line treatment in Japan until recently when it started to be supplemented with glucose lowering medications as well [44–46]. In China, the majority of oral anti-diabetic drugs belong to the Sulfonylureas class. This is the oldest of the anti-diabetic drug classes and the majority of hypoglycemic medicines on China’s 2012 EDL are within this category in spite of the availability of new classes [47–51]. Sulfonylureas has consistently been the first line treatment for T2D in Korea, with no competition until 2010, when Metformin started getting popular in the Korean market and its consumption and sales increased by 2013 [52].

In contrast to the Asian populations, we found that the genes identified in the Finnish, European, and American populations are mostly related to insulin resistance. The Saudi population is also characterized, as well as many Arab countries, by Adipocyte dysfunction which associates obesity to insulin resistance and diabetes [53]. Research shows that Metformin is the first line treatment for T2D in USA, UK, Finland and Saudi Arabia [54–57]. In fact Metformin has been on the list of the top ten prescription drugs in the USA for years, ranking four in 2018 and 2019 [58] and on the list of top 100 most prescribed drugs in the UK [59] and ranks fifth on the list of most prescribed drugs in Saudi Arabia from 2010 to 2015 [60].

In 2017, Metformin had the highest average antidiabetic drug prescriptions per physician in Finland which also falls under the top 10 most commonly prescribed medicine categories [61]. The Finnish Medicine Agency – Fimea [62] estimates what proportion of the population theoretically receives Metformin; in terms of drug daily dose per 1000 inhabitants per day. The Fimea 2017 report shows that Metformin consumption is highest among all antidiabetic drugs between 2014 and 2017 (31.65, 31.65,30.95 and 31.61 respectively with an increasing gap with Sulfonylureas; the latter shows a decreasing daily consumption of 3.94, 3.15, 2.38 and 1.76 for the same 4 years respectively). Metformin has also been associated with a good change in the gut microbiota, which improves insulin sensitivity [63].

T2D has a heterogeneous and multifactorial etiology, with many associated factors including gut microbiome, and possibly genetic subtypes that are yet to be uncovered. Although T2D treatments work at different degrees of efficiency from one person to another, the above analysis confirms previous research [64, 65] indicating that, of the currently known T2D subtypes, certain subtypes seem to be most common in certain ethnicities, and that Asian populations are more characterized by decrease in insulin secretion capacity as opposed to American, European, and other Caucasian populations which have insulin resistance as the most common reason for T2D [66, 67]. Also, the network-based T2D subtype analysis (Fig. 5) shows more overlap between the two subtypes of T2D than the gene-based analysis, which supports our results and previous findings [68].

The experimental results we obtained using prostate cancer data show consistency to T2D results in the sense that more statistically significant overlap is realized as we go through the pipeline from SNP to network overlap analysis, which supports our hypothesis and demonstrates the generalizability of the methods.

## Conclusions

In this work, we developed computational algorithms and statistical frameworks to assess the functional overlap between disease-associated variants in different populations, expecting that the variants identified in certain populations correspond to similar biological processes. For this purpose, we developed a multi-layered framework, where genomic loci, protein-coding genes, biological pathways in which these proteins are active, and networks of physical and functional interactions between these proteins are systematically evaluated for potential overlap.

Our results, show that the overlap between different populations grow as the level of abstraction coarsens from genomic location to biological function. More interestingly, we were able to show that differences in the biological processes that are implicated in different populations align with the targets of first-line treatments of T2D in each population. We were also able to assess the generalizability of our method by testing its applicability to another complex disease. To this end, our results represent an innovative and potentially significant tool for preventing, curing, and treating disease, in that population-specific functional annotation of disease-associated genes can be used to design personalized treatment strategies in precision medicine.

It is important to note that the results presented here do not conclusively show a causal link between the genomic markers identified via GWAS and the first-line T2D treatments in these populations. Establishment of such a link would require further quantitative analysis to understand whether specific types of diabetes II are over represented in specific populations, and whether the medicine that was prescribed for each patient was appropriate. Furthermore, the prevalence of a specific kind of medicine in a country may not be related to the etiology of disease, but can rather be due to historical or political reasons. Further research is required to answer these questions.

## Methods

### Overlap matrices and cumulative overlap functions

The objective of this study is to characterize the functional overlap between loci that are identified to be significantly associated with a complex disease based on samples from different populations. To address this problem, we assume that we are given a collection L = *{L*_*1*_*, L*_*2*_*, …, L*_*k*_} of sets of genomic loci identified to be associated with the disease across *k* populations (in this study, we have *k =* 9), such that the set *L*_*i*_ contains the loci that are found to be significant based on the samples obtained in the *i*th population. Based on this information, we compute five overlap matrices and five cumulative overlap functions. Each overlap matrix is a *k* × *k* matrix that represents the pairwise overlap between the disease-associated loci in pairs of populations based on a certain notion of functional overlap. Each cumulative overlap function is a function in the form *f*:{1, *…, k*} → [0,1], assessing the fraction of biological entities (individual loci, loci in LD, genes, functions, subnetworks) that are found to be associated with the disease in at least a given number of the populations.

We compute the following overlap matrices:

1. SNP Overlap Matrix [*Σ*_*ij*_]_kxk_ assesses the overlap between the loci that are found to be associated with the disease in populations *i* and *j,* where *k* is the number of populations.
2. LD SNP Overlap Matrix [*Λ*_*ij*_]_kxk_ assesses the overlap between the loci that are found to be associated with the disease in populations *i* and *j,* such that two loci are considered to be overlapping if they are in linkage disequilibrium.
3. Gene Overlap Matrix [*Γ*_*ij*_]_kxk_ assesses the overlap between genes that harbor loci that are found to be associated with the disease in populations *i* and *j.*
4. Functional Overlap Matrix [*Φ*_*ij*_]_kxk_ assesses the overlap between the biological processes that are enriched in genes harboring loci that are found to be associated with the disease in populations *i* and *j.*
5. Network Overlap Matrix [*Ν*_*ij*_]_kxk_ assesses the overlap between the subnetworks of protein-protein interaction networks that are enriched in genes harboring loci that are found to be associated with the disease in populations *i* and *j.*

In the following discussion, we explain how we compute each of these overlap matrices. The notation used in this section is provided in Table 3.

**Table 3:** Notation Used in this Paper

*S*	Set of tagSNPs
*S*_*i(j)*_	Set of tagSNPs in population i(j)
*L*_*ij*_	Set of tagSNPs in population i that have significant LD partners in population j
*L*_*ji*_	Set of tagSNPs in population j that have significant LD partners in population i
*G*_*i(j)*_	Set of genes in population i(j)
*F*_*i(j)*_	Set of functions in population i(j)
*Ν*_*i(j)*_	Set of genes constituting most significant subnetwork in population i(j)
*S*_*k*_	Set of tagSNPs that are significant in at least K populations
*L*_*k*_	Set of tagSNPs that have significant LD partners in at least K populations
*G*_*k*_	Set of genes that are associated with at least K populations
*F*_*k*_	Set of functions that are associated with at least K populations
*N*_*k*_	Set of genes, constituting the most significant networks, that are associated with at least K populations

#### SNP overlap matrix

We define the SNP Overlap Ratio *Σ*_*ij*_ between two populations *i* and *j* as the Jaccard coefficient of the overlapping SNPs between the populations, i.e., the fraction of common significant tagSNPs in the two populations among the number of significant tagSNPs in the two populations:

$$ {\varSigma}_{ij}=\mid {S}_i\cap \kern0.5em {S}_j\mid \kern0.5em /\kern0.5em \mid {S}_i\kern0.5em \cup {S}_j\mid $$

Where *S*_*i*_ and *S*_j_ denote the sets of tagSNPs that are found to be significantly associated with the disxeases in populations i and j, respectively.

In order to quantify the overall overlap between the *k* populations, we define the *cumulative SNP overlap* function *σ*(*l)* for *1* ≤ *l* ≤ *k* as follows:

$$ \sigma\ (l)=\mid {S}_l\mid /\mid S\mid $$

Where *S*_*l*_ denotes the set of tagSNPs that are found to be significantly associated with the disease in at least *l* populations*.* Observe that 0 ≤ *σ*(*l)* ≤ *1, σ*(*1) = 1*, and *σ*(*l)* is a monotonically non-increasing function of *l.* All cumulative overlap functions we define below also exhibit these properties.

Since several probes map to the same SNP, while computing the sets *S*_*i*_ for 1 ≤ i ≤ k, we first remove duplicate SNP lists in every population. For the same reason, we also compute the overlap ratio for a pair of populations as the number of common tagSNP over the number of all unique tagSNPs in the populations combined.

#### LD SNP overlap matrix

We define the linkage disequilibrium (LD) SNP overlap ratio *Λ*_*ij*_ between two populations *i* and *j* as the fraction of common significant tagSNPs in the two populations that have significant LD partners in the other population among the number of significant  tagSNPs in the two populations :

$$ {\varLambda}_{ij}=\mid {L}_{ij}\cup {L}_{ji}\mid \kern0.5em /\kern0.5em \mid {S}_i\cup {S}_{\mathrm{j}}\mid $$

Where *L*_*ij*_ denotes the set of tagSNPs that are found to be significantly associated with the diseases in population *i* and have significant LD partners in the other population *j*, and *L*_*ji*_ denotes the set of tagSNPs that are found to be significantly associated with the diseases in population *j* and have significant LD partners in the other population *i*.

In order to quantify the overall overlap between the *k* populations, while using LD to expand the definition of tagSNPs, we define the *cumulative LD SNP overlap* function *λ(l)* for 1 ≤ *l* ≤ *k* as follows:

$$ \lambda (1)=\mid {\mathrm{L}}_{\mathrm{l}}\mid /\mid \mathrm{S}\mid $$

Where *L*_*l*_ denotes the set of tagSNPs that have LD partners found to be significantly associated with the disease in at least *l* populations*.*

To find SNPs that are in linkage disequilibrium (LD), we input each population’s tagSNPs into SNPsnap [69] for LD search using HapMap3 release 2 [70] dataset. For this purpose, we use the European panel (CEU) for the European and American populations (W and D), European and Toscani in Italia (TSI) for the French and Lebanese populations, and the Japanese and Chinese panels (JPT + CHP) for Finland and the Asian populations. We consider two SNPs to be in LD if they have an *r*^*2*^ of at least 0.5 and they are within 500Kbs of each other on the genome.

#### Gene overlap matrix

We define the Gene Overlap Ratio *Γ*_*ij*_ between two populations *i* and *j* as the fraction of common genes that harbor significant tagSNPs (in a region of interest of 100 kb upstream and 10 kb downstream) in the two populations among the number of genes in the two populations, i.e.:

$$ {\varGamma}_{ij}=\mid {G}_i\cap {G}_j\mid /\mid {G}_i\cup {G}_j\mid $$

Where *G*_*i*_ and *G*_*j*_ denote the sets of genes that harbor significant tagSNPs that are found to be significantly associated with the diseases in populations *i* and *j*, respectively.

In order to quantify the overall overlap between the *k* populations, we define the *cumulative gene overlap* function *γ(l)* for 1 ≤ *l* ≤ *k* as follows:

$$ \gamma (l)=\mid {G}_l\mid \kern0.5em /\mid G\mid $$

Where *G*_*l*_ denotes the set of genes that harbor significant tagSNPs that are found to be significantly associated with the disease in at least *l* populations*.*

In order to map SNPs to Genes, all Refseq transcripts [71] for hg38 assembly are downloaded from UCSC Table browser and extended 100 kb upstream and 10 kb downstream. Refseq IDs are translated to HGNC symbols. SNPs are mapped to their positions on hg38 assembly through biomaRt [72] and are intersected with gene coordinates. Such intersections result in gene lists matching each population.

#### Functional overlap matrix

We define the Functional Overlap Ratio *Φ*_*ij*_ between two populations *i* and *j* as the fraction of common biological processes that are enriched in genes harboring significant tagSNPs (+ 100 kb/− 10 kb) in populations *i* and *j* among the number of biological processes that are enriched in genes harboring significant tagSNPs (+ 100 kb/− 10 kb) in the two populations, i.e.:

$$ {\varPhi}_{ij}=\mid {F}_{\mathrm{i}}\cap {F}_{\mathrm{j}}\mid /\mid {F}_{\mathrm{i}}\cup {F}_{\mathrm{j}}\mid $$

Where *F*_i_ and *F*_j_ denote the sets of significant biological processes in populations *i* and *j*, respectively.

In order to quantify the overall overlap between the *k* populations, we define the *cumulative functional overlap* function *ϕ(l)* for *1* ≤ *l* ≤ *k* as follows:

$$ \phi (l)=\mid {F}_l\mid /\mid F\mid $$

Where *F*_*l*_ denotes the set of biological processes that are found to be significantly associated with the disease in at least *l* populations*.*

To identify biological processes that are enriched in T2D-associated SNPs for each population, we use enrichment analysis of gene sets using the WebGestalt R package [73, 74]. Biological Process terms are used and False Discovery Rate (FDR) threshold is set to 5%.

#### Network overlap matrix

For each population, we feed the loci that are found to be associated with the disease to the Prix-Fixe [20] network analysis tool. The Prix-Fixe tool uses a network based disease-associated subnetwork identification algorithm that uses genome-scale shared function networks to identify the most functionally coherent subnetwork of genes spanning the disease associated loci. Using shared function networks as a reference, the algorithm evaluates gene combinations, constraining the choice to one gene from each disease associated locus, for their shared function, hence similar to choosing compatible food items from a prix fixe restaurant menu, with one dish from each course. The algorithm outputs a list of genes, with their disease association scores, such that the top scored genes constitute the most coherent disease associated subnetwork in each population.

We define the Network Overlap Ratio *Ν*_*ij*_ between two populations *i* and *j* as the fraction of common genes constituting subnetworks of protein-protein interaction networks that are enriched in genes harboring loci that are found to be associated with the disease in populations *i* and *j*, among all genes in populations *i* and *j*, i.e.:

$$ {N}_{\boldsymbol{i}\boldsymbol{j}}=\mid {\boldsymbol{N}}_{\boldsymbol{i}}\mathbf{\cap}{\boldsymbol{N}}_{\boldsymbol{j}}\mid /\mid {\boldsymbol{N}}_{\boldsymbol{i}}\cup {\boldsymbol{N}}_{\boldsymbol{j}}\mid $$

Where *N*_*i*_ and *N*_*j*_ denote the sets of genes constituting the most significant subnetworks in populations *i* and *j* respectively.

In order to quantify the overall overlap between the *k* populations, we define the *cumulative network overlap* function *ν (l)* for *1* ≤ *l* ≤ *k* as follows:

$$ \nu\ (l)=\mid {N}_l\mid /\mid N\mid $$

Where *N*_*l*_ denotes the set of genes constituting the subnetworks that are found to be most significantly associated with the disease in at least *l* populations*.*

### Permutation tests

To test the statistical significance of our results, we create a thousand simulated data sets at each level of genomic granularity (SNPs, LD SNPs, Genes, Functions, Networks) with a simulated set size for each population matching that of the original data set. A thousand permutations are done and in each permutation, permuted overlap functions are calculated as described above. This results in 1000 overlap function vectors for *k* = 1 to 9 corresponding to each permuted data set. For each *k,* in each permuted set, the minimum, maximum and mean values of the overlap function across all permutations are calculated. These calculations are then used to visualize distribution of the overlap function in permuted datasets and assess the significance of the observed overlap based on this distribution.

### Permutation tests for SNP overlap

In order to perform the permutation tests for SNP overlap, we prepare a SNP pool. For each study, the genotyping array model is identified. Array annotation files are downloaded from UCSC genome annotation database [75]. Further, SNPs from WTCCC and dbGAP populations are extracted and pooled together with SNPs from all arrays, this resulted in 2,028,276 unique SNPs. (Note: Imputation data is not used, since these studies do not provide the final imputation results after quality control). All the populations together with their SNPs constitute a SNP set, which is used as a pool for permutation tests. SNP sets are then randomly sampled, with replacement across populations, such that the size of each set matches that of the observed SNP set for each population.

### Permutation tests for LD-SNP overlap

The SNP sets generated in each iteration of permutation tests for SNP overlap are provided to SNPsnap [69] tool for LD SNP extraction using the parameters used for the original data set.

### Permutation tests for gene overlap

A gene pool is created from all human genes; all Refseq transcripts [71] for hg38 assembly are downloaded from UCSC Table browser and extended 100 kb upstream and 10 kb downstream and Refseq IDs are translated to HGNC symbols. All HGNC symbols matching Refseq IDs are combined and duplicates removed. The final HGNC list served as the pool for random gene sampling. In each of the thousand permutations, gene sets are randomly sampled from the gene pool, with replacement across populations, matching the size of the observed gene set.

### Permutation tests for functional overlap

Enrichment analysis is done for one thousand randomly sampled gene sets. In each permutation enrichment analysis is performed with the same parameters as the actual enrichment analysis. At this point, two populations (L and Fr) were not enriched in any biological processes, so the two populations were dropped from the analysis and further analysis is done on *k* = 7 populations only.

### Permutation tests for network overlap

The SNP sets generated in each of the permutation tests for SNP overlap are provided to Prix-Fixe [20] network analysis tool, which outputs the most coherent disease associated subnetwork for each population. This results in 1000 most coherent disease associated subnetworks for each population.

## Abbreviations

dbGaP: Database of genotypes and phenotypes
FDR: False discovery rate
GWAS: Genome-wide association studies
LD SNP: SNPs in linkage disequilibrium
SNP: Single nucleotide polymorphism
T2D: Type II diabetes
WTCCC: Wellcome-Trust Case Control Consortium

## References

[1] Rosenberg NA, Huang L, Jewett EM, Szpiech ZA, Jankovic I, Boehnke M (2010). Genome-wide association studies in diverse populations. Nat Rev Genet.

[2] Cooper RS, Tayo B, Zhu X (2008). Genome-wide association studies: implications for multiethnic samples. Hum Mol Genet.

[3] Need AC, Goldstein DB (2009). Next generation disparities in human genomics: concerns and remedies. Trends Genet.

[4] Soundararajan U, Yun L, Shi M, Kidd KK (2016). Minimal SNP overlap among multiple panels of ancestry informative markers argues for more international collaboration. Forensic Sci Int Genet.

[5] Burton PR, Clayton DG, Cardon LR, Craddock N, Deloukas P (2007). Genome-wide association study of 14,000 cases of seven common diseases and 3,000 shared controls. Nature..

[6] Mailman MD, Feolo M, Jin Y, Kimura M, Tryka K, Bagoutdinov R (2007). The NCBI dbGaP database of genotypes and phenotypes. Nat Genet.

[7] Scott LJ, Mohlke KL, Bonnycastle LL, Willer CJ, Li Y, Duren WL (2007). A genome-wide association study of type 2 diabetes in Finns detects multiple susceptibility variants. Science.

[8] Sabatti C, Hartikainen AL, Pouta A, Ripatti S, Brodsky J, Service SK (2009). Genome-wide association analysis of metabolic traits in a birth cohort from a founder population. Nat Genet.

[9] Rung J, Cauchi S, Albrechtsen A, Shen L, Rocheleau G, Cavalcanti-Proenca C (2009). Genetic variant near IRS1 is associated with type 2 diabetes, insulin resistance and hyperinsulinemia. Nat Genet.

[10] Al-Daghri NM, Alkharfy KM, Alokail MS, Alenad AM, Al-Attas OS, Mohammed AK (2014). Assessing the contribution of 38 genetic loci to the risk of type 2 diabetes in the Saudi Arabian population. Clin Endocrinol.

[11] Al-Daghri NM, Al-Attas OS, Krishnaswamy S, Mohammed AK, Alenad AM, Chrousos GP (2015). Association of Type 2 diabetes mellitus related SNP genotypes with altered serum adipokine levels and metabolic syndrome phenotypes. Int J Clin Exp Med.

[12] Gosadi IM (2016). Assessment of the environmental and genetic factors influencing prevalence of metabolic syndrome in Saudi Arabia. Saudi Med J.

[13] Ban HJ, Heo JY, Oh KS, Park KJ (2010). Identification of type 2 diabetes-associated combination of SNPs using support vector machine. BMC Genet.

[14] Kwak SH, Kim SH, Cho YM, Go MJ, Cho YS, Choi SH (2012). A genome-wide association study of gestational diabetes mellitus in Korean women. Diabetes..

[15] Yamauchi T, Hara K, Maeda S, Yasuda K, Takahashi A, Horikoshi M (2010). A genome-wide association study in the Japanese population identifies susceptibility loci for type 2 diabetes at UBE2E2 and C2CD4A-C2CD4B. Nat Genet.

[16] Imamura M, Takahashi A, Yamauchi T, Hara K, Yasuda K, Grarup N (2016). Genome-wide association studies in the Japanese population identify seven novel loci for type 2 diabetes. Nat Commun.

[17] Ma RC, Hu C, Tam CH, Zhang R, Kwan P, Leung TF (2013). Genome-wide association study in a Chinese population identifies a susceptibility locus for type 2 diabetes at 7q32 near PAX4. Diabetologia..

[18] Shu XO, Long J, Cai Q, Qi L, Xiang YB, Cho YS (2010). Identification of new genetic risk variants for type 2 diabetes. PLoS Genet.

[19] Ghassibe-Sabbagh M, Haber M, Salloum AK, Al-Sarraj Y, Akle Y, Hirbli K (2014). T2DM GWAS in the Lebanese population confirms the role of TCF7L2 and CDKAL1 in disease susceptibility. Sci Rep.

[20] Tasan M, Musso G, Hao T, Vidal M, MacRae CA, Roth FP (2015). Selecting causal genes from genome-wide association studies via functionally coherent subnetworks. Nat Methods.

[21] Cantley J, Ashcroft FM (2015). Q&a: insulin secretion and type 2 diabetes: why do beta-cells fail?. BMC Biol.

[22] Prasad RB, Groop L (2015). Genetics of type 2 diabetes-pitfalls and possibilities. Genes..

[23] Schumacher FR, Al Olama AA, Berndt SI, Benlloch S, Ahmed M, Saunders EJ (2018). Association analyses of more than 140,000 men identify 63 new prostate cancer susceptibility loci. Nat Genet.

[24] Al Olama AA, Kote-Jarai Z, Berndt SI, Conti DV, Schumacher F, Han Y (2014). A meta-analysis of 87,040 individuals identifies 23 new susceptibility loci for prostate cancer. Nat Genet.

[25] Benafif S, Kote-Jarai Z, Eeles RA (2018). A review of prostate Cancer genome-wide association studies (GWAS). Cancer epidemiology, biomarkers & prevention : a publication of the American Association for Cancer Research, cosponsored by the American society of preventive. Oncology..

[26] Benafif S, Eeles R (2016). Genetic predisposition to prostate cancer. Br Med Bull.

[27] Eeles RA, Kote-Jarai Z, Giles GG, Olama AA, Guy M, Jugurnauth SK (2008). Multiple newly identified loci associated with prostate cancer susceptibility. Nat Genet.

[28] Beuten J, Gelfond JA, Martinez-Fierro ML, Weldon KS, Crandall AC, Rojas-Martinez A (2009). Association of chromosome 8q variants with prostate cancer risk in Caucasian and Hispanic men. Carcinogenesis..

[29] Cheng I, Chen GK, Nakagawa H, He J, Wan P, Laurie CC (2012). Evaluating genetic risk for prostate cancer among Japanese and Latinos. Cancer epidemiology, biomarkers & prevention : a publication of the American Association for Cancer Research, cosponsored by the American Society of Preventive. Oncology..

[30] Takata R, Akamatsu S, Kubo M, Takahashi A, Hosono N, Kawaguchi T (2010). Genome-wide association study identifies five new susceptibility loci for prostate cancer in the Japanese population. Nat Genet.

[31] Wang M, Takahashi A, Liu F, Ye D, Ding Q, Qin C (2015). Large-scale association analysis in Asians identifies new susceptibility loci for prostate cancer. Nat Commun.

[32] Wu Y, Chen H, Ji Y, Na R, Mo Z, Ye D (2018). Validation of the novel susceptibility loci for prostate cancer in a Chinese population. Oncol Lett.

[33] Marzec J, Mao X, Li M, Wang M, Feng N, Gou X (2016). A genetic study and meta-analysis of the genetic predisposition of prostate cancer in a Chinese population. Oncotarget..

[34] Chen R, Ren S, Sun Y (2013). Genome-wide association studies on prostate cancer: the end or the beginning?. Protein Cell.

[35] Oh JJ, Lee SJ, Hwang JY, Kim D, Lee SE, Hong SK (2017). Exome-based genome-wide association study and risk assessment using genetic risk score to prostate cancer in the Korean population. Oncotarget..

[36] Han Y, Rand KA, Hazelett DJ, Ingles SA, Kittles RA, Strom SS, et al. Prostate Cancer susceptibility in men of African ancestry at 8q24. J Natl Cancer Inst. 2016;108(7):djv431. 10.1093/jnci/djv431 PMC4948565 26823525

[37] Hilal L, Shahait M, Mukherji D, Charafeddine M, Farhat Z, Temraz S (2015). Prostate Cancer in the Arab world: a view from the inside. Clin Genitourin Cancer.

[38] Mayo Clinic Staff. Type 2 diabetes: Mayo Foundation for Medical Education and Research; 2018 [updated September 15. Available from: http://www.mayoclinic.org/diseases-conditions/type-2-diabetes/diagnosis-treatment/treatment/txc-20169988.

[39] Marin-Penalver JJ, Martin-Timon I, Sevillano-Collantes C, Del Canizo-Gomez FJ (2016). Update on the treatment of type 2 diabetes mellitus. World J Diabetes.

[40] Canadian Institutes of Health Research. Metformin: ClinCalc LLC; 2018 [Accessed on December 12]. Available from: https://www.drugbank.ca/drugs/DB00331.

[41] Canadian Institutes of Health Research. Gliclazide: ClinCalc LLC; 2018 [Accessed on December 12]. Available from: https://www.drugbank.ca/drugs/DB01120.

[42] Canadian Institutes of Health Research. Tolbutamide: ClinCalc LLC; 2018 [Accessed on December 12]. Available from: https://www.drugbank.ca/drugs/DB01124.

[43] Canadian Institutes of Health Research. Acetohexamide: ClinCalc LLC; 2018 [Accessed on December 12]. Available from: https://www.drugbank.ca/drugs/DB00414.

[44] Fukushima M, Suzuki H, Seino Y (2004). Insulin secretion capacity in the development from normal glucose tolerance to type 2 diabetes. Diabetes Res Clin Pract.

[45] Kuroe A, Fukushima M, Usami M, Ikeda M, Nakai Y, Taniguchi A (2003). Impaired beta-cell function and insulin sensitivity in Japanese subjects with normal glucose tolerance. Diabetes Res Clin Pract.

[46] Oishi M, Yamazaki K, Okuguchi F, Sugimoto H, Kanatsuka A, Kashiwagi A (2014). Changes in oral antidiabetic prescriptions and improved glycemic control during the years 2002-2011 in Japan (JDDM32). J Diabetes Invest.

[47] Wilsdon T, Li L (2015). Assessing the value of treatment for diabetes to patients, the healthcare system, and wider society – a case study on China.

[48] Tian X, Song Y, Zhang X (2012). National Essential Medicines List and policy practice: a case study of China's health care reform. BMC Health Serv Res.

[49] CFDA. China Food and Drug Administration (CFDA): CFDA; n.d. 2012. [Available from: http://eng.sfda.gov.cn/WS03/CL0755/.

[50] Israeli Foreign Trade Administration. Israel Global Blogs Network: Israel Ministry of Economy and Industry; n.d. 2013. [Available from: http://itrade.gov.il/china-en/2013/04/23/national-essential-medicine-list-2012-edition-released.

[51] Pan C, Xing X, Han P, Zheng S, Ma J, Liu J (2012). Efficacy and tolerability of vildagliptin as add-on therapy to metformin in Chinese patients with type 2 diabetes mellitus. Diabetes Obes Metab.

[52] Korean Diabetes Association. Diabetes Fact Sheet In Korea 2018: KDA; 2018 [updated October 10. Available from: http://www.diabetes.or.kr/bbs/index.html?sub_menu=&code=e_resource&category=1&gubun=&page=1&number=381&mode=view&order=&sort=&keyfield=&key=.

[53] Abuyassin B, Laher I (2015). Obesity-linked diabetes in the Arab world: a review. East Mediterr Health J.

[54] Jarvinen S, Laine MK, Eriksson JG (2016). Comparison of use of diabetic medication and clinical guidelines in four Nordic countries. Ann Med.

[55] National Institute for Health and Care Excellence (2017). Type 2 diabetes in adults: management: NICE.

[56] Grygotis L. Updated guideline for oral pharmacologic treatment of type 2 diabetes: Clinical Advisor; 2017 [updated January 4. Available from: https://www.clinicaladvisor.com/diabetes-resource-center/updated-guideline-for-pharmacologic-treatment-of-t2d/article/629636/.

[57] Alhreashy FA, Mobierek AF (2014). Prescription practice for diabetes management among a female population in primary health care. Int J Family Med.

[58] ClinCalc DrugStats Database. Metformin Hydrochloride - Drug Usage Statistics, United States, 2006 - 2016: ClinCalc LLC; 2018 [updated July 19. Available from: https://clincalc.com/DrugStats/Drugs/MetforminHydrochloride.

[59] Bodell M. Top 100 Most-Prescribed Medications in UK Hospitals: Nursing Notes; 2018 [updated April 8. Available from: https://nursingnotes.co.uk/the-100-most-common-medications-in-uk-hospitals/.

[60] AlKhamees OA, AlNemer KA, Bin Maneea MW, AlSugair FA, AlEnizi BH, Alharf AA (2018). Top 10 most used drugs in the Kingdom of Saudi Arabia 2010–2015. Saudi Pharm J.

[61] Social Insurance Institution of Finland. Statistical database Kelasto: KELA; 2010 [Accessed on December 12]. Available from: https://www.kela.fi/web/en/statistical-database-kelasto_contents#Sickness.

[62] Finish Medicines Agency. Drug consumption in 2014–2017: FIMEA; 2018 [accessed on December 12].Available from: http://raportit.nam.fi/raportit/kulutus/laakekulutus_e.htm.

[63] Doheny K, Escobar JS, Apovian C. Metformin alters microbiota, improving insulin sensitivity: endocrine web; 2018 [Accessed on December 12]. Available from: https://www.endocrineweb.com/professional/type-2-diabetes/metformin-alters-microbiota-improving-insulin-sensitivity.

[64] Yoon KH, Lee JH, Kim JW, Cho JH, Choi YH, Ko SH (2006). Epidemic obesity and type 2 diabetes in Asia. Lancet (London, England).

[65] Min HK (1996). Non-insulin-dependent diabetes mellitus (NIDDM) in Korea. Diabet Med.

[66] Ma RC, Chan JC (2013). Type 2 diabetes in east Asians: similarities and differences with populations in Europe and the United States. Ann N Y Acad Sci.

[67] Guilherme A, Virbasius JV, Puri V, Czech MP (2008). Adipocyte dysfunctions linking obesity to insulin resistance and type 2 diabetes. Nat Rev Mol Cell Biol.

[68] Bakir-Gungor B, Sezerman OU (2013). The identification of pathway markers in intracranial aneurysm using genome-wide association data from two different populations. PLoS One.

[69] Pers TH, Timshel P, Hirschhorn JN (2015). SNPsnap: a web-based tool for identification and annotation of matched SNPs. Bioinformatics..

[70] Altshuler DM, Gibbs RA, Peltonen L, Altshuler DM, Gibbs RA, The International HapMap Consortium (2010). Integrating common and rare genetic variation in diverse human populations. Nature..

[71] O'Leary NA, Wright MW, Brister JR, Ciufo S, Haddad D, McVeigh R (2016). Reference sequence (RefSeq) database at NCBI: current status, taxonomic expansion, and functional annotation. Nucleic Acids Res.

[72] Durinck S, Moreau Y, Kasprzyk A, Davis S, De Moor B, Brazma A (2005). BioMart and Bioconductor: a powerful link between biological databases and microarray data analysis. Bioinformatics (Oxford, England).

[73] Zhang B, Kirov S, Snoddy J (2005). WebGestalt: an integrated system for exploring gene sets in various biological contexts. Nucleic Acids Res.

[74] Wang J, Duncan D, Shi Z, Zhang B (2013). WEB-based GEne SeT AnaLysis toolkit (WebGestalt): update 2013. Nucleic Acids Res.

[75] Rosenbloom KR, Armstrong J, Barber GP, Casper J, Clawson H, Diekhans M (2015). The UCSC genome browser database: 2015 update. Nucleic Acids Res.

